# *Ginkgo biloba*: A Treasure of Functional Phytochemicals with Multimedicinal Applications

**DOI:** 10.1155/2022/8288818

**Published:** 2022-02-28

**Authors:** Noor-E-Tabassum, Rajib Das, Mashia Subha Lami, Arka Jyoti Chakraborty, Saikat Mitra, Trina Ekawati Tallei, Rinaldi Idroes, Amany Abdel-Rahman Mohamed, Md. Jamal Hossain, Kuldeep Dhama, Gomaa Mostafa-Hedeab, Talha Bin Emran

**Affiliations:** ^1^Department of Pharmacy, Faculty of Pharmacy, University of Dhaka, Dhaka 1000, Bangladesh; ^2^Department of Biology, Faculty of Mathematics and Natural Sciences, Sam Ratulangi University, Manado 95115, North Sulawesi, Indonesia; ^3^The University Centre of Excellence for Biotechnology and Conservation of Wallacea, Institute for Research and Community Services, Sam Ratulangi University, Manado 95115, North Sulawesi, Indonesia; ^4^Department of Pharmacy, Faculty of Mathematics and Natural Sciences, Universitas Syiah Kuala, Kopelma Darussalam, Banda Aceh 23111, Indonesia; ^5^Department of Forensic Medicine and Toxicology, Zagazig University, Zagazig 44511, Egypt; ^6^Department of Pharmacy, State University of Bangladesh, 77 Satmasjid Road, Dhanmondi, Dhaka 1205, Bangladesh; ^7^Division of Pathology, ICAR-Indian Veterinary Research Institute, Izatnagar, Bareilly 243122, Uttar Pradesh, India; ^8^Pharmacology Department, Health Sciences Research Unit, Medical College, Jouf University, Sakaka, Saudi Arabia; ^9^Department of Pharmacy, BGC Trust University Bangladesh, Chittagong 4381, Bangladesh

## Abstract

*Ginkgo biloba* is an ancient plant species that is thought to provide a variety of health benefits to living organisms and contains plenty of bioactive components, making it a chemically diversified plant. *G. biloba* has been shown to have a variety of medicinal and pharmacological properties, including anticancer, antidementia, antidiabetic, antiobesity, antilipidemic, antimicrobial, antioxidant, antilipid peroxidation, antiplatelet, anti-inflammatory, hepatoprotective, antidepressant, antiaging, immunomodulatory, antihypertensive, and neuroprotective effects and is frequently used to treat neurological, cardiovascular, and respiratory diseases, such as tardive dyskinesia. Therefore, this review described the therapeutic applications of *G. biloba*. In addition to describing the therapeutic potential, this review also evaluates the chemical constituents, toxicity, adverse effect, synergistic effect, and the clinical studies of this plant which have been utilized for therapeutic benefits but have demonstrated other consequences. The capacity of *G. biloba* components to act as free radical scavengers is critical, and combining its extract with other plant extracts has been shown to synergistically boost antioxidant properties. *G. biloba* used long-term or at high doses that resulted in some adverse effects. Severe drug interactions have also been reported in both animals and humans when combined with other medications. The available data established from both preclinical and clinical studies confirm the potential of *G. biloba* plant extract in various diseases. Besides, the safety and efficacy of *G. biloba* continue to require verification through additional experimentation to guide medicinal use.

## 1. Introduction

Plant-based phytochemicals have been utilized for over 1000 years and serve as a major platform for novel drug discovery. *Ginkgo biloba* L. (family: Ginkgoaceae; English name: Maidenhair tree) is a key source of novel herbal medications containing many bioactive constituents with therapeutic efficacy. This plant species is ancient, deciduous, tall, and strong, with fan-shaped, irregularly lobed leaves, growing to heights of up to 40 meters [1]. The genus name “biloba” refers to the tree's two separate lobes, and the genus name Ginkgo is a phonetic translation of the tree's Japanese name. *G. biloba* is clearly classified within the plant kingdom, and this plant is often termed a “living fossil” because, evolutionarily, it is one of the oldest seed plants [2]. After the atomic bomb detonation in Hiroshima, Japan, in 1946, *G. biloba* was the first plant to germinate. Microorganisms, chemical pollutants, insects, and environmental factors have a little effect on species survival [2].

*G. biloba* has been used as a traditional medicinal plant for longer than 2000 years in China and other parts of the world [3] and is currently grown in Europe, Asia, Argentina, North America, and New Zealand [4]. Ample research investigations focusing on this plant have shown promising therapeutic benefits [5–10], such as the treatment of asthma, tuberculosis (TB), skin problems, stomach discomfort, bronchitis, hearing loss, nervousness, arteriosclerosis, thrombus formation, ischemic heart disease, and diabetes mellitus (DM) [5]. Combining *G. biloba* extract (GBE) with grape seed skin extracts, quercetin, green tea, resveratrol, and bilberry extracts has been shown to reduce diastolic blood pressure in hypertensive individuals [5]. In addition to antihypertensive effects, the GBE EGb 761 appears to display antioxidant properties [6, 7]. The ability of EGb 761 to scavenge free radicals produced by reactive nitrogen species (RNS) and reactive oxygen species (ROS) is a critical contributor to the medicinal effects of this extract [8, 9]. When EGb 761 is combined with other plant extracts, the antioxidant capabilities are often synergistically enhanced. Promising chemical compounds that have been identified in this plant include flavonoids (quercetin, kaempferol, and isorhamnetin), terpenoids (bilobalide and ginkgolides), bioflavonoids (ginkgetin, sciadopitysin, and isoginkgetin), and organic acids (ginkgolic acid), which can affect a variety of biological processes [11]. As a result, *G. biloba* leaf extract is widely utilized to treat cardiovascular and neurological disorders [3, 12] and is one of the most commonly used traditional medicinal plants. Best health supplements available in the market that contain *G. biloba* are Nutricost *Ginkgo biloba*, GreeNatr Panax Ginseng *G. biloba* tablets, Nature's Bounty *G. biloba*, and VH Nutrition *G. biloba* [13, 14].

In recent years, clinical studies examining the efficacy and safety of herbal medicines and investigations into their underlying pharmacological functions have received a lot of attention. However, studies profiling the biopharmaceutical characteristics of herbal medicines, including efficacy and safety, are less common [15–19]. Plant-based medical products are expected to meet the same potency, purity, and biological content standards for efficacy that have been established for chemically defined synthetic medicines and dietary supplements, which are often not satisfied [15].

The fundamental aim of this review is to discuss the dissemination of *G. biloba*, including trade patterns, and provide an overview of the bioactive compounds found in this plant, including therapeutic effects, the molecular pathways thought to mediate these effects, nutritional values, the potential for toxicity, and interactions with other medications and food supplements.

## 2. Chemical Constituents

Several chemical compounds have been derived from *G. biloba* with a wide range of therapeutic activities. In recent years, novel chemical compounds, including new terpenoids and lignans, have been identified in *G. biloba* [20], as described in the following sections.

### 2.1. Flavonoids

**Figure 1:**
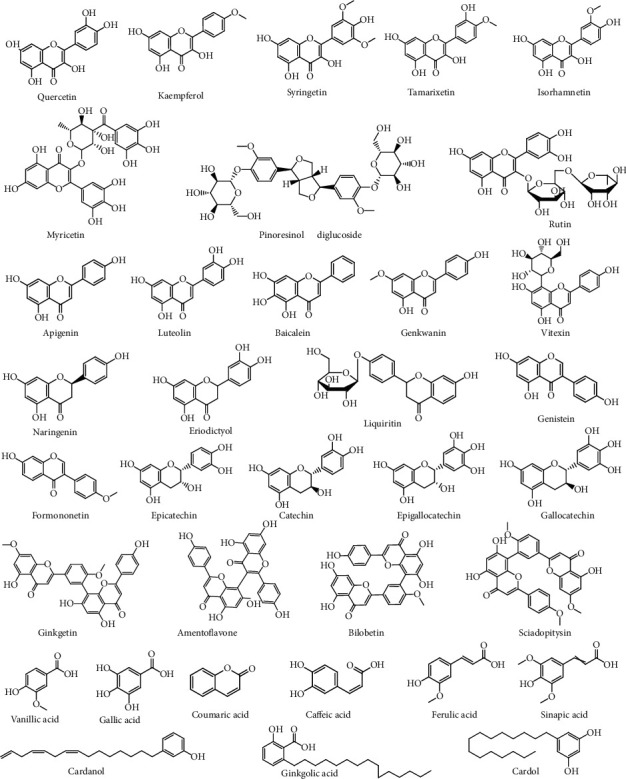
Chemical structures of flavonol, flavonoids (flavone, flavanone, isoflavone, flavan-3-ols, and biflavonoids), alkylphenols, alkylphenolic acids, carboxylic acids, and lignans.

Liquid chromatography-mass spectrometry (LC-MS) has been used to identify and separate several flavonoids found in *G. biloba* [21–25]. To date, 110 flavonoids have been identified, including kaempferol 3-O-*α*-l-[6000-p-coumaroyl(*β*-d)-glucopyranosyl(1,2)-rhamnopyranoside]-7-O-*β*-d-glucopyranoside and isorhamnetin 3-O-*α*-l-[6000-p-coumaroyl(*α*-d)-glucopyranosyl(1,2)-rhamnopyranoside], which were identified in an n-BuOH extract of *G. biloba* leaves. These flavonoids exhibit antioxidant properties when they bind with six other flavonol glycosides: quercetin 3-O-*β*-D-glucopyranoside, quercetin 3-O-*β*-rutinoside, quercetin 3-O-*α*-L-[6‴-p-coumaroyl-(*β*-D)-glucopyranosyl-(1,2)-rhamnopyranoside], kaempferol 3-O-*α*-L-[6‴-p-coumaroyl-(*β*-D)-glucopyranosyl-(1,2)-rhamnopyranoside], quercetin 3-O-*β*-D-glucopyranosyl-(1–2)-alpha-L-rhamnopyranoside, and quercetin 3-O-*α*-L-[6‴-p-coumaroyl-(*β*-D)-glucopyranosyl-(1,2)-rhamnopyranoside]-7-O-*β*-D-glucopyranoside [26].  Figure 1 shows the flavonol structures and other flavonoids. Flavonoids can be characterized into seven groups: flavanones, isoflavones, flavone, biflavones, flavan-3-ols, flavonols, and biginkgosides. Ma et al. first separated and described biginkgosides in 2016, describing the isolation of nine biginkgosides [27].

### 2.2. Terpenoids

Ten diterpenoid lactones have been discovered, known as ginkgolides Q, P, N, M, L, K, J, C, B, and A. Until recently, bilobalide was thought to be the only sesquiterpene lactone in *G. biloba*, but Dong et al. announced a new bilobalide isomer in 2020 [28]. *G. biloba* also contains nor-terpenoids, including three nor-sesquiterpenoids discovered by Shu et al. in *G. biloba* L. [29]. The chemical structures of terpenoids are shown in Figure 1.

### 2.3. Alkylphenols and Alkylphenolic Acids

Alkylphenols can be divided into five groups: cardols, cardanols, *α*-hydroxycardanols, urushiols, isourushiols, and alkylphenolic acids. These chemical constituents of *G. biloba* are shown in Figure 1 [30]. Although ginkgolic acids are known to be toxic [31], they have also been reported to display potential pharmacological effects.

### 2.4. Carboxylic Acids

The carboxylic acids that have been identified in *G. biloba* include ferulic acid, p-coumaric acid, protocatechuic acid, caffeic acid, p-hydroxybenzoic acid, m-hydroxybenzoic acid, vanillic acid, isovanillic acid, gallic acid, and sinapic acid [32], the structures of which are shown in Figure 1. Phenolic acids have been demonstrated to form glycosidic or covalent bonds, with the exception of the free form in GBL [20].

### 2.5. Lignans

Lignans were identified in *G. biloba* roots in 2015 and in *G. biloba* seeds in 2018 [33, 34]. Lignans obtained from *G. biloba* show antioxidant properties [33]. In 2018, lignans were also discovered in GBE [20]. Pinoresinol contains 0.012–0.020 mg/mL diglucoside and 1.05–1.87 mg/mL total lignan glycosides. Five lignans were isolated from *G. biloba* by Shu et al. [29], as shown in Figure 1.

### 2.6. Proanthocyanidins

Prodelphinidin and procyanidin are two proanthocyanidins that have been identified in *G. biloba* at a ratio of 85 : 15. Prodelphinidin is an epigallocatechin polymer, whereas procyanidin is comprised of epicatechin [20].

### 2.7. Polyprenols

Polyprenols, which are active ingredients identified in *G. biloba*, comprised of long chains of 14–24 isopentenyl units and have a similar structure as S-polyterpene alcohol (dolichols), which can be found in mammals, including people [35].

### 2.8. Polysaccharides

Okhti et al. examined the chemical structures of polysaccharides found in *G. biloba* and discovered that they were composed of glucose, rhamnose, mannose, arabinose, and galactose [36].

### 2.9. Others

The toxic component 4′-O-methylpyridoxine (MPN) was first discovered in *G. biloba* seeds and isolated by Klein et al. [37]. *G. biloba* essential oil contains 68 compounds, which includes 42.11% sesquiterpenes [38].

## 3. Nutraceutical Value of *Ginkgo biloba*

Nutraceuticals (also known as phytochemicals or functional foods) are bioactive chemical compounds found in nature that have beneficial properties, such as promoting health, preventing illness, or other medicinal effects. Nutraceuticals are used in a wide range of products produced by the pharmaceutical industry, the recently combined pharmaceutical/agribusiness industry, the food industry, and the herbal and dietary supplement sector [39]. *G. biloba* products were among the most widely sold medicinal products by US health food retailers, according to Blumenthal in 2000. Unlike ginkgo leaves, *Ginkgo* nuts have long been used as food and medicine and were first referenced in the herbals in approximately 1350 AD [40]. *G. biloba* seeds contain high levels of vitamin C, carbohydrates, riboflavin, proteins, and various other nutrients. *Ginkgo* nuts are thought to offer health benefits, such as cancer prevention and neurologic disease treatment [41]. *G. biloba* seeds are an integral food crop and can be used in cakes, glazed vegetables, drinks, and alcohols, including various delicacies and sweets, depending on the cooking process used. Popular meals in China include *Ginkgo-*steamed egg and *Ginkgo*-fried chicken, and Cheng Teng is a classic sweet treat found in Southeast Asia. *G. biloba* nuts have been served as a side dish in Japan since the Edo era (1600–1867) [42]. *G. biloba* seeds are also commonly used as conventional Chinese medical supplements for the prevention of fever, cough, and sputum production and to treat skin disorders, gonorrhea, toothache, and overactive bladder. *G. biloba* seeds are listed in the Chinese traditional medical reference Compendium of Materia Medica, and *G. biloba* seeds are commonly recommended as a functional food for the prevention of neurodegenerative diseases. However, the presence of ginkgolic and allergy-inducing acids, such as MPN and ginkgotoxin glucoids, can lead to vitamin B6 deficiencies and other unwanted side effects, preventing *G. biloba* products from becoming everyday foods [43]. Researchers have begun exploring methods for reducing levels of toxic substances found in *G. biloba* nuts using various processing methods. The incubation of *G. biloba* nuts in 3 volumes (v/w) of 5 g/L Na_2_CO_3_ at 15°C for 3 h was found to effectively remove ginkgolic acid from *G. biloba* nuts [44]. In addition, traditional food processing procedures, such as baking, boiling, and heating by microwaves, can also minimize seed toxicity. Because *G. biloba* seeds can be eaten as food and purchased freely in markets, the literature examining the bioactivities, manufacturing methods, and modified pharmacological functions of *G. biloba* seeds typically examine the use of seeds as food additives rather than as supplemental tablets or capsules (Table 1).

## 4. Bioactive Compounds in *Ginkgo biloba*

**Figure 2:**
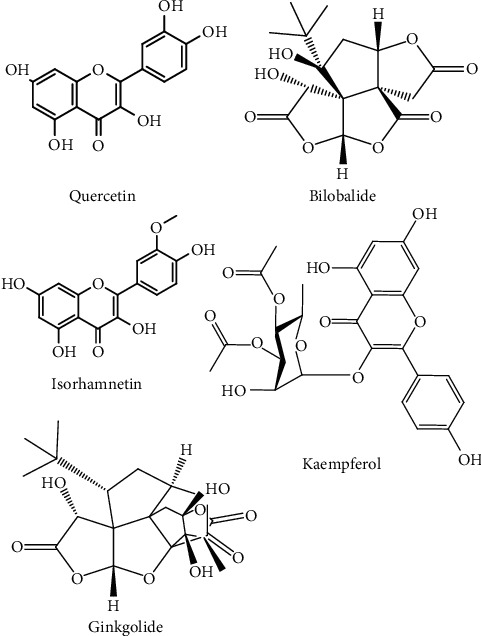
Chemical structures of *Ginkgo biloba*'s key bioactive compounds.

*G. biloba* leaves have been extensively investigated as a source of the plant's major medicinal components. Active chemical components found in *G. biloba* leaves include flavonoids and terpenoids, and plant extracts have exhibited a variety of pharmacological activities, including antibacterial, antioxidant, anti-inflammatory, antiallergic, and cytotoxic anticancer activities [4, 11]. Many other bioactive compounds, including bioflavonoids, organic acids, and polyprenols, have been identified in *G. biloba* (Table 2). Other constituents of *G. biloba* with known pharmacological activities are ginkgolides and bilobalide. Ginkgolides can be divided into five types (A, B, C, J, and M), in which each have a unique set of properties. Flavonoids, such as quercetin, kaempferol, and isorhamnetin, are found as glycoside derivatives in *G. biloba* (Figure 2). A standardized leaf extract of *G. biloba*, known as EGb 761, includes 6% terpenoids, 5%–24% flavonoid glycosides, 10% organic acids, and other bioactive compounds that are known to exert a wide range of beneficial health effects [4, 11].

## 5. Pharmacological Activities

*G. biloba* is a traditional medicinal herb with a variety of therapeutic properties. GBE is commonly used to treat asthma and bronchitis [59], and the leaves and nuts of this plant have been utilized as Chinese traditional medicine for millennia. *G. biloba* leaves have traditionally been used to treat heart issues and cure skin infections, whereas the nuts have traditionally been used to treat various types of respiratory illnesses, including asthma, chest pain, and cough, in addition to bladder irritation and alcoholism [60]. *G. biloba* has been shown to effectively treat symptoms associated with Alzheimer's disease (AD), epilepsy, cerebrovascular illness, stroke, and peripheral vascular disease [61, 62]. Evidence suggests that GBE exerts a variety of pharmacological activities, such as lowering the risk of cardiovascular disease, preventing ischemia-induced oxidation [63, 64], increasing cerebral blood flow [65], hepatoprotective functions [66, 67], and platelet antagonization [68]. GBEs have also been studied for anticancer effects, although this remains a poorly understood area. The whole extract derived from *G. biloba* fruit provides better effects for various indications than any specific isolated compounds [69].

### 5.1. Treatment of Respiratory Diseases

Acute respiratory distress syndrome (ARDS), asthma, and chronic obstructive pulmonary disease (COPD) are all associated with airway inflammation, and disease onset, progression, and prognosis are all considered to be influenced by inflammatory processes. Airway inflammation is exacerbated by neutrophils and other inflammatory cells. Under inflammatory conditions, neutrophils mediate two fundamental metabolic reactions: respiratory burst and chemotaxis. Wu et al. suggested that during lipopolysaccharide (LPS)-induced pulmonary injury, ginkgolide M (GM) and ginkgolide B (GB) were equally effective for reducing the aggregation of inflammatory cells, such as lymphocytes, neutrophils, and macrophages, and improving cytological lung damage [70]. According to Tao et al., the production of interleukin (IL)-4, IL-5, IL-6, IL-8, IL-13, and tumor necrosis factor (TNF)-*α* decreased considerably when allergy model mice were treated with EGb 761. EGb 761 also regulates the activity of leukocyte elastase, a protein involved in blood coagulation disorders, pulmonary damage, and chronic bronchitis, and the components of EGb 761 were able to reduce inflammation in the lungs [71].

### 5.2. Anticancer Effects

Cancer is a disease in which certain cells in the body begin to proliferate uncontrollably, eventually spreading to other areas of the body. Cancer is a global problem, and according to the National Institutes of Health, in 2016, 1.7 million new cancer patients were diagnosed with cancer in the United States alone, with 0.6 million people dying as a result of the disease, indicating the importance of developing novel cancer therapies. Ahmed et al. investigated the effectiveness of GBE for the treatment of rats with hepatocellular carcinoma (HCC). GBE significantly enhanced the histological characteristics of liver tissue, decreasing the levels of alpha-fetoprotein (AFP), glypican-3 (GPC-3), and carcinoembryonic antigen (CEA) in HCC rats via the increased gene expression of inhibitor of growth (ING)-3 and the decreased gene expression of the transcription factor forkhead box protein 1 (FOXP1) in the liver [72]. Another study conducted by Han et al. demonstrated the anti-Lewis lung cancer (LLC) efficacy of *G. biloba* exocarp extracts (GBEE), which limited in vitro LLC cell growth by regulating catenin and Wnt3a expression in a dose-dependent manner, and GBEE demonstrated similar effects on the expression of various proteins, including Wnt3a, vascular endothelial growth factor (VEGF), and phosphorylated (p)-protein kinase B (Akt)/Akt, when administered to C57BL/6J mice. GBEE decreased the protein expression levels of p-Akt/Akt, VEGF, VEGF receptor (VEGFR), and *β*-catenin, contributing to the inhibition of lung carcinoma metastasis. VEGF and VEGFR2 mRNA levels were similarly reduced, suggesting that GBEE suppresses tumor angiogenesis, resulting in antitumor effects. The underlying mechanisms of these outcomes were associated with the inhibition of the Wnt, catenin, and VEGF signaling pathways in LLC [73]. Qian et al. investigated the impacts of GBE on human SGC7901 gastric cancer cells in vitro and in vivo. GBE inhibited the development of SGC7901 gastric cancer cells in a dose and time-dependent manner, and GBE increased the number of cells in the G1 stage while decreasing the number of cells in the S stage, as assessed by flow cytometry. GBE-treated cells showed reduced mRNA and protein levels of cyclin D1 and c-Myc, indicating that GBE might inhibit cell cycle progression by reducing cyclin D1 and c-Myc production [74]. When Liu and colleagues used EGb 761 in stomach cancer cells, they observed similar effects; however, the underlying mechanisms of EGb 761 activity were restricted to the kinase suppressor of Ras 1 (KSR1)-mediated extracellular signal-regulated kinase (ERK) 1/2 pathway [44].

### 5.3. Antidementia Effects

Most EGb 761 clinical trials are targeted at enhancing cognition and memory, with some concentrating on dementia, particularly dementia presentations associated with the gradual loss of cognition and memory, including vascular dementia, Lewy body dementia, and frontotemporal dementia. Because EGb 761 can prevent amyloid production and toxicity, control excitotoxic glutamatergic neurotransmission, and act as a radical scavenger, it could potentially be used to treat multiple dementia etiologies [75].

### 5.4. Antidiabetic Effects

DM is a complicated disease that is currently estimated to affect 366 million individuals globally, which is predicted to increase to 552 million affected individuals by 2030 [76]. Several in vivo investigations have demonstrated that GBE has antidiabetic effects. Bilobalide, according to one study, shielded lipid cells against oxygen deficiency-induced insulin resistance and reduced inflammation by increasing adiponectin secretion, blocking the serine phosphorylation of insulin receptor substrate 1 (IRS-1) receptors in the insulin signaling cascade, lowering inflammatory adipokine production, and decreasing nuclear factor kappa B (NF-*κ*B)/c-Jun N-terminal kinase (JNK) activation [77]. According to these findings, ginkgolide B (orally administered at 5 mg/kg/day) enhanced cholinergic vasorelaxation and phenylephrine vasoconstriction in male Sprague Dawley rats. Ginkgolide B also boosted superoxide dismutase (SOD) and endothelial nitric oxide (NO) synthase (eNOS) activity while decreasing H_2_S production and malonaldehyde (MDA) levels. Ginkgolide B also improved the expression of cystathionine-lyase proteins, cystathionine-synthetase, NADP-oxidase subunits, and the glutathione peroxidase (GPX1) enzyme [35].

### 5.5. Antiobesity Effects

High-fat diets contribute to the pathogenesis of obesity by altering the regulation of the peripheral metabolism and food consumption at the level of the central nervous system (CNS), leading to weight gain, increased insulin tolerance, and other metabolic disorders [59]. GBE administration was found to be beneficial for reducing body weight. Food consumption over a 24-hour period was measured by assessing the difference between the quantity provided and quantity remaining. The gene expression levels of insulin receptor (IR), IL-10, and lipid receptor R1 (Adipo R1) and the phosphorylation of Akt all increased significantly, potentially stimulating the insulin signaling cascade. By contrast, TNF-*α* levels and NF-*κ*B p65 phosphorylation were reduced. The anti-inflammatory activities of GBE, which reduces TNF-*α* levels in retroperitoneal fat deposits, can mitigate the negative effects associated with excessive high-fat diet consumption [78]. The likely mediators of these antiobesity effects are the GBE components ginkgetin, isoginkgetin, bilobetin, and sciadopitysin, which have moderate to strong inhibitory effects on pancreatic lipase (PL), with half-maximal inhibitory concentration (IC_50_) values ranging from 2.90 M to greater than 12.78 M, indicating that the compounds found in *G. biloba* may serve as lead compounds for the development of bioflavonoid-type PL inhibitors [79]. The impacts of ginkgolide B on hepatic steatosis and body weight were studied in a high-fat diet-induced model of obesity in C57BL/6 male mice, which revealed that treatment with ginkgolide B (0.1% per day) induced human pregnane *X* receptor (hPXR) mRNA expression in the liver in a dose-dependent manner, without affecting the expression of peroxisome proliferator-activated receptor (PPAR), liver *X* receptor (LXR), farnesyl *X* receptor (FXR), or LXR trans-activities. Ginkgolide B also reduced plasma triglyceride (TG) levels and body weights while resolving hepatic steatosis [80]. Treatment with 3–100 M ginkgolide C substantially decreased lipid aggregation in an in vitro study of HepG2 liver cells and increased TG degradation by increasing the phosphorylation of hormone-sensitive lipase and the production of adipose TG lipase. Ginkgolide C was found to enhance lipolysis and reduce lipid aggregation through the activation of the sirtuin 1 (SIRT1)–AMP-activated protein kinase (AMPK) cascade in an oleic acid-induced model of the fatty liver [80].

### 5.6. Antilipidemic Effect

Dyslipidemia is defined by high TG levels, low high-density lipoprotein cholesterol (HDL-C) levels, and increased low-density lipoprotein cholesterol (LDL-C) levels. In addition, hyperlipidemia has been associated with obesity and insulin resistance. In a separate study of male rabbits, *G. biloba* (10 mg/kg/day) treatment dramatically decreased plasma cholesterol and TG levels while significantly increasing HDL-C levels compared with the control group. Furthermore, in aortic tissue, *G. biloba* has been found to lower MDA levels while raising glutathione (GSH) levels [81].

### 5.7. Treatment of Cardiovascular Diseases

**Figure 3:**
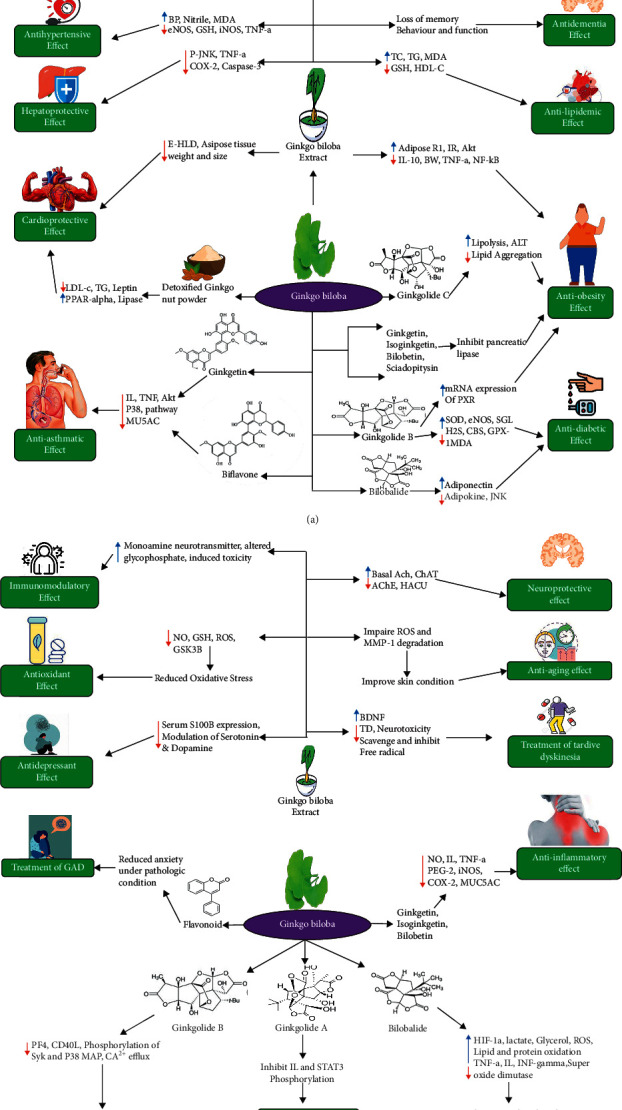
(a), (b) Therapeutic potential of *Ginkgo biloba*.

The impacts of *G. biloba* seeds on the cholesterol metabolism were initially studied by Mahadevan et al., who studied the effects of the lipid metabolism and cardiovascular disease prevention mediated by various components of *G. biloba* seeds, including whole seeds, water-soluble fraction, and lipid-soluble fraction [60]. In vitro studies have shown that *G. biloba* seeds can impact the synthesis of apolipoprotein B and LDL receptor, modulating the blood cholesterol level of the liver. In vivo, whole seeds reduced hepatic cholesterol levels while increasing serum cholesterol levels. The lipid-soluble portion reduced hepatic cholesterol levels, whereas the water-soluble portion increased serum cholesterol levels, suggesting that lipid-soluble portion of *G. biloba* seeds could be used to lower the risks of heart disease. *Ginkgo* seed ethanol extract was shown to be beneficial in high-fat diet-fed mice. The ethanolic extract effectively prevented fat accumulation and lowered the overall body weight in mice, associated with a substantial decrease in adipocyte size and epididymal adipose tissue weight compared with untreated animals [40], suggesting that *Ginkgo* seed ethanol extract might reduce serum levels of HLD cholesterol. These data indicate that *G. biloba* seeds have a hypocholesterolemic impact via the reduction of lipid synthesis, which might be beneficial for the treatment of cardiovascular disorders [82]. Liu et al. examined the effects of *G. biloba* nut powder on the lipid metabolism in obese mice. The liver weights and serum and hepatic levels of LDL cholesterol, leptin, TG, and total cholesterol in obesity model mice fed *G. biloba* nut powder were equivalent to those of control mice but substantially lower than those in the untreated model group. They also indicated that detoxified *G. biloba* nut powder might decrease damage and enhance the lipid metabolism [83]. The therapeutic potentials of *G. biloba* are shown in Figures 3(a)-3(b)).

### 5.8. Antimicrobial Effects

Antimicrobial activity has been established for several components of *G. biloba* plants, including phenolic acids, polysaccharides, and proteins. Ginkgolic acids have been the subject of several experiments examining the antimicrobial functions of *G. biloba* seeds. Ginkgolic acids are fatty acids with the compositions C13 : 0, C15 : 0, C15 : 1, C17 : 1, and C17 : 2, and the antimicrobial activities of ginkgolic acids are aided by the presence of an alkyl side-chain [40]. Ginkgolides have been shown to be bactericidal against *Staphylococcus aureus*, *Bacillus cereus*, and *Bacillus subtilis*, but not against *Escherichia coli*. Ginkgolic acids, by contrast, have demonstrated potent antibacterial activity against *E. coli*, with a minimum inhibitory concentration (MIC) of 7.5 × 10^−3^ g/mL. Ginkgolides also demonstrated antimicrobial activity against *Penicillium* and *B. subtilis*, with MIC values of 2.5 × 10^−4^ g/mL and 1.5 × 10^−4^ g/mL, respectively. Wu et al. identified a protein with broad-spectrum antibacterial activity in *G. biloba* plants using an ammonium sulfate precipitation. Some Gram-negative and Gram-positive bacteria were inhibited by the identified protein, with the strongest activity against *Klebsiella pneumoniae*, with an MIC of 1.0 × 10^−5^ g/mL. An MIC of 1.0 × 10^−5^ was also identified against *Torulaspora delbrueckii*, *Penicillium*, and *Aspergillus niger*. Polysaccharides comprised 50%–80% of the ethanol precipitation of *G. biloba* seeds, and 87.4% of polysaccharides and 1.0% of proteins displayed antibacterial characteristics [84].

### 5.9. Antioxidant Effects

Tissues undergo DNA oxidative stress, oxidative protein injury, lipid peroxidation, and other oxidative injuries when they mature. Neurological activity, sensory tissues, and the cardiovascular system may all be affected as a result [84], and these defects can have significant influences on the progression of degenerative diseases [85]. *G. biloba* has several distinct antioxidant components, making it difficult to test each antioxidant independently. In diseased tissues, higher quantities of free radicals and oxidatively damaged lipids, DNA, and proteins and reduced levels of antioxidants have all been reported. Terpenes, flavonoids, and bioflavonoid components are thought to contribute the antioxidant capabilities of *G. biloba*, which has been shown to be beneficial against a variety of free radical-generating substances, including oxyferryl, superoxide, hydroxyl, NO, and peroxyl radicals. The antioxidant properties of *G. biloba* contribute to protecting the cardiovascular system, brain, and retina from free radical damage associated with aging. The antioxidant capabilities and flavonol contents of *G. biloba* leaf crude extracts were investigated [86], and Kaur et al. found that ginkgolide B derived from EGb 761 applied to human neuroblastoma IMR-32 and SHSY5Y cells reduced ROS/RNS production by prooxidant A25–35 peptides [86]. MDA and NO levels increased with age but were suppressed by pretreatment with EGb 761. By controlling oxidative stress, EGb 761 can protect against ischemic injury [87]. Aydin utilized cisplatin to promote oxidation in the rat brain and observed that EGb 761 reduced GSH and NO levels in brain tissue, which reduced oxidative stress [7].

### 5.10. Wound Healing

Wound healing is a biochemical mechanism that repairs weakened tissues and preserves skin integrity. Hemostasis, inflammation, proliferation, and remodeling are the four phases of skin wound healing. Cytokines promote healing through various methods, including boosting basement membrane material production, avoiding dehydration, enhancing inflammation, and hastening the formation of granulated tissues [88]. Okumus et al. examined the antioxidant impacts of *G. biloba* on the prevention of radiation-induced cataract development in the rat lens. In the presence of radiation, GBE significantly enhanced total (enzymatic and nonenzymatic) superoxide scavenger activity (TSSA), glutathione-S-transferase (GST), glutathione reductase (GRD), and nonenzymatic superoxide scavenger activity (NSSA). In the irradiation + GBE groups, the activity of lens xanthine oxidase (XO) was reduced substantially [89]. In cranium-irradiated studies, *G. biloba* increased the activities of SOD and GPX while decreasing lipid peroxidation activity [90]. Frostbite occurs when tissues are exposed to cold temperatures, commonly resulting in freezing on the extremities and can result in the need for amputation or cause tissue failure and impairment, associated with the development of free radicals, which cause additional tissue damage. Dong reported that EGb 761 treatment was able to reduce frostbite damage as a free radical scavenger [91]. The in vivo and in vitro studies examining the therapeutic effects of *G. biloba* are given in Table 3.

### 5.11. Antiplatelet Activity

Platelet-activating factor (PAF) is a phospholipid-derived messenger that contributes to the immunological response to infection and neuronal damage induced by ischemia and excitotoxic stress. PAF is produced in response to inflammatory damage and can serve as a paracrine, endocrine, or autocrine messenger by activating the PAF receptor (PAFR) to activate inflammatory proteins [75]. The PAFR is associated with the Janus kinase (JAK)/signal transducer and activator of the transcription (STAT) signaling pathway, which regulates gene transcription in response to cytokines and growth factors [5]. The release of CD40L and regulated upon activation, normal T cell expressed and presumably secreted (RANTES) by platelets activated by thrombin and collagen is significantly reduced by treatment with 0.6 mg/mL ginkgolide B. In thrombin (0.5 U/mL)-activated platelets, ginkgolide B (0.6 mg/mL) suppressed ATP release by 50.8% and partially reduced calcium efflux by 52.7%, according to a recent study. Ginkgolide B was reported to reduce platelet release by decreasing the phosphorylation of p38 mitogen-activated protein kinase (MAPK) and Syk [97]. As a PAF antagonist, ginkgolide C was found to be 25 times less effective than ginkgolide B due to the presence of the 7-OH replacement, which is absent from other ginkgolides. Ginkgolide C had lower area under the curve (AUC) and peak serum concentration (*C*_max_) than the other ginkgolides and is methylated in vivo much faster than the other EGb 761 components, which might explain the overall reduced activity [97]. In addition to its impact on PAF, bilobalide, like ginkgolides, offers additional health benefits. Bilobalide's anti-inflammatory effects are supported by a new study that shows it reduces hypoxia-induced inflammation and discomfort [98].

### 5.12. Anti-Inflammatory Effects

Inflammation is a complicated biochemical process that leads to the destruction of tissue homeostasis. Inflammation can be induced by various molecular, mechanical, or physical agents and can be classified as chronic or acute [106]. Inflammation is typically triggered by the release of substances from tissues and migratory cells, such as histamine, prostaglandins (PGs), leukotrienes (LTs), bradykinin, PAF, and IL [107]. Various cytokines and chemokines produced by dendritic cells and macrophages stimulate circulating leukocytes after they enter the local injury site. Neutrophils phagocytose antigens, releasing ROS and cytokines, include IL-1, IL-6, and TNF-*α* [108]. Polyphenolic chemicals found in *G. biloba*, such as flavonoids, have been shown to have anti-inflammatory properties [109]. Coughs, asthma, and other lung disorders have long been treated with *G. biloba* in traditional Chinese medicine. In an ovalbumin-induced allergy mouse model, the pharmacological effects of several GBEs were examined. The ethyl acetate phase of the extract included anti-inflammatory compounds, which were identified using high-performance liquid chromatography (HPLC)-MS. The primary compounds were identified as biflavones, one of which ginkgetin has a good fit score with leukocyte elastase, and the effects of biflavones were studied in both in vivo and in vitro settings. In human neutrophil elastase (HNE)-stimulated A549 cells, ginkgetin significantly decreased the abnormal expression of the Akt and p38 pathways. Mucin 5AC (MUC5AC) mRNA expression was also reduced by biflavones in HNE-stimulated A549 cells and the allergic mouse modes, and biflavone-treated mice showed reduced levels of inflammatory cells and cytokines. These data suggested that *G. biloba* biflavones may decrease leukocyte elastase activity, and *G. biloba* has been identified as a functional food for the treatment of airway inflammation [71]. In another study, LPS-induced RAW264.7 macrophages treated with 100 g/mL of the ethanol extract of *G. biloba* flowers or the chloroform and ethyl acetate fractions showed greatly reduced prostaglandin E2 (PGE2), NO, and IL-6 outputs. Bilobetin and isoginkgetin, the most powerful compounds identified in GBEs, increased the NO inhibition ratios to 80.19% and 82.37% at 50 M, respectively. They also inhibited TNF-*α*, IL-6, PGE2, and cyclooxygenase 2 mRNA levels in a dose-dependent manner, indicating that they may be promising candidates for the discovery of new anti-inflammatory drugs [103].

### 5.13. Hepatoprotective Effects

The antioxidant activity of *G. biloba* is thought to be responsible for its hepatoprotective benefits, associated with the restoration of SOD, GPX, and catalase (CAT) activities, in addition to increasing glutathione contents and reducing the levels of lipid peroxidation and hydroperoxides in the liver [110]. The hepatoprotective effect of GBEs in rats with obstructive yellowing was also examined [69, 111], revealing that GBE greatly improved serum transaminase levels and reduced liver histological damage, resulting in increased liver defense. Pretreatment with GBE prevented the release of TNF-*α* and upregulation of IL-6 mRNA. In addition, the necrotic region of the central lobe was diminished by GBE therapy [69, 112]. *G. biloba* has hepatoprotective effects against carbon tetrachloride (CCl_4_)-induced hepatic oxidative injury in rats [113]. *G. biloba* phytosome (GBP) may act through an initial decrease in hepatic peroxidative activities, accompanied by the inhibition of glutathione (GSH)-related enzymes [67]. The antioxidant and hepatoprotective properties of GBE have been shown to prevent liver fibrosis in rats [114].

### 5.14. Antidepressant Effects

Depression is a widespread and long-lasting mental disorder that affects mood and feelings, resulting in global economic losses. Gut microbiota, especially probiotics, have been shown to influence the development of depression and recovery. Polysaccharides have antidepressive effects through the regulation of the microbiota-gut-brain axis [110, 115]. GBE can effectively reduce depressive symptoms and suppress serum S100B expression, which suggests that GBE restores the neurological activity in older adults treated for. A synergistic effect is observed when GBE is combined with antidepressant drugs, resulting in more rapid results than the use of single antidepressant drugs alone [116]. A water-soluble *G. biloba* polysaccharide reduces depression caused by stress and reverses intestinal dysbiosis. GPS mice had higher serotonin and dopamine levels than the unpredictable chronic moderate stress mice in many brain areas. The polysaccharides derived from *G. biloba* leaves may represent successful pharmacological candidates for depression treatment [117]. GBE resulted in a strong increase in venlafaxine in PSD treatment [118]. Diterpene ginkgolides (DGs) in GBE have been shown to exert neuroprotective effects across many previous studies. DGs have antidepressant but not antianxiety effects in mice, indicating that GBE might be useful for the treatment of major depressive disorders [105]. Chronic DG therapy greatly improves the characteristics of depression [119]. In mice with heart disease, GBE increased antidepressant-like activities. TNF-*α*, IL-1, and 5-HT levels in the hippocampus were decreased after GBE administration. GBE also inhibited serotonin release in peripheral blood and activated hypoxia-inducible factor 1 (HIF-1)-associated antiapoptotic pathways [120]. Compared with control groups, pretreatment with 30 and 60 mg/kg/day GBE substantially reduced depressive-like effects in RSS rats and prevented changes in DNA methylation and protein expression of the brain-derived neurotrophic factor (BDNF) in the hippocampus induced by persistent stress. These results indicate that GBE may exert antidepressant effects by modulating BDNF expression in the hippocampal nucleus [121].

### 5.15. Antiaging Effects

Skin ages primarily due to the effects of oxidative stress. Aging skin, increased collagen loss, and poor desquamation can be distinguished by increased wrinkles and a dry and dull appearance. *G. biloba* leaf extract and aqueous *G. biloba* ethanol extract have been shown to display skin protective effects, and the molecular pathways involved have been studied using HaCaT keratinocytes, revealing antioxidant effects and antiaging results [122]. Many different GBEs have been shown to cause biphasic reactions in a wide variety of cell types. GBE applied as a topical formula achieved good skin penetration and retention, with beneficial effects for the skin, including protection from ultraviolet (UV) damage [123]. EGb 761 treatment in rats showed a preventive function against frostbite and likely alleviated reperfusion damage by minimizing tissue peroxidation [91], suggesting that EGb 761 may serve as an antiaging agent. However, the antiaging effects of EGb 761 remain inconclusive, particularly the effects on the CNS [124]. Total lactones of *Ginkgo* (TLG) applied to aged mice displayed antiaging effects by attenuating lipid peroxidation, NO levels, and cerebral apoptosis [125]. The main components of EGb 716 can regulate numerous pathways and exert antiaging effects by inhibiting inflammation, reducing oxidative stress, and improving insulin resistance, mediated by several targets, such as PPARG, DPP4, and GSK3B [126].

### 5.16. Immunomodulatory Effects

Immunomodulators regulate the immune system. Most herbs defend the body against infection through two basic mechanisms: destroying pathogens or increasing immunity [127]. *G. biloba* polysaccharides (GBPS) exert enhanced antitumor and immune responses. GBPS-2 and GBPS-3 greatly improved macrophage phagocytosis and encouraged NO, TNF-*α*, IL-1, and IL-6 activity, suggesting that GBPS-2 and GBPS-3 may serve as useful food supplements for boosting immunity [128]. One study examined the immunostimulatory effects of dietary *G. biloba* (GB) on oxidative stress and toxicity induced by the organophosphate insecticide diazinon. Plasma total immunoglobulin, lysozyme activity, and peroxidase activity were substantially higher in non-diazinon-exposed fish 60 days of feeding with 1 and 2 g GB/kg. In a group fed 0.5 g GB/kg, pulmonary burst activity and complement activity were considerably enhanced. Throughout the feeding study, peroxidase activity, immunoglobulin activity, and lysozyme activity were considerably decreased in the group fed with 4 g GB/kg. No substantial changes in renal IL-1*β* or transforming growth factor beta-1 (TGF*β*-1) gene expression were observed between the non-GB-supplemented fish and GB-supplemented fish. Both immunological components were reduced significantly in diazinon-exposed fish fed with and without 0.5 and 4 g GB/kg diets. No differences in immunity components were observed for fish fed 1 and 2 g GB/kg diets during the course of the experiment. Diazinon exposure increased the expression of IL-1*β* and TGF*β*-1 genes in fish fed 0.5 and 4 g GB/kg diets. These findings indicated that GB displayed an immunogenic effect against diazinon toxicity at optimal dietary levels (1-2 g GB/kg), but high dietary levels (4 g GB/kg) resulted in immunosuppressive effects, suggesting that dietary levels must be optimized to ensure beneficial effects [102]. *G. biloba* exocarp polysaccharides (GBEP) were found to exert effects on cellular immunity and humoral immunity in a mouse immunosuppressive model [129]. Therefore, GBE can be a rapid, healthful, and somewhat effective therapy for halting disease progression and increasing immunity when intake levels are optimized [130].

### 5.17. Treatment of Tardive Dyskinesia

Tardive dyskinesia (TD) is a common adverse reaction to antipsychotic drugs, which are commonly used to treat dementia and other serious psychiatric illnesses. TD is characterized by uncontrollable, stiff, jerky facial and body motions, resulting in involuntary movements, such as blinking one's eyes, sticking out one's tongue, or waving one's arms [131]. Although low BDNF has been indicated in the pathophysiology of TD, the etiology remains inconclusive, and no well-accepted treatments have been developed. The administration of EGb 761 results in increased BDNF levels through antioxidant mechanisms, which have neuroprotective effects, suggesting that EGb 761 could potentially reduce TD symptoms [132]. A randomized controlled trial (RCT) was performed to assess the antioxidant and free radical-scavenging properties of EGb 761, in which the Abnormal Involuntary Movement Scale was used to quantify the severity of TD symptoms as the primary outcome. EGb 761 (240 mg/day) outperformed the placebo for minimizing the incidence of TD symptoms. A meta-analysis revealed that adjunctive EGb 716 appears to be an efficient and secure alternative for improving TD in schizophrenia patients. However, further RCTs remain necessary to demonstrate the efficacy and protective abilities of adjunctive EGb 716, particularly in terms of protecting the cognitive capacity, in cases of TD [133]. EGb 761 appears to be an appropriate therapy for reducing TD effects in schizophrenic patients, which is likely mediated by the well-known antioxidant activity of the extract [134].

### 5.18. Treatment of Generalized Anxiety Disorder

Generalized anxiety disorder (GAD) is marked by constant and excessive worry over a variety of topics. Individuals who suffer from GAD expect the occurrence of constant catastrophic events that manifest as excessive concern regarding income, health, family, jobs, and other matters. Individuals who suffer from GAD often struggle to contain their worries [135]. Globally, anxiety and mental illness are the most commonly diagnosed conditions, and various medications have been used to treat mental conditions, including dementia, depression, and anxiety disorders, including GBE and other *G. biloba* products, which are used globally [136]. The literature suggests that GBE can offer beneficial effects on ischemia, hypoxia, and stress reduction in cases of cognitive deterioration [137, 138]. EGb 761 improves emotional function and stabilizes mood in older adult patients with cognitive impairment. In addition, EGb 761 has been shown to relieve anxiety symptoms among people with mental deterioration, including in younger patients with anxiety symptoms [139]. The chemical structures and synergy of various chemical components found in EGb 761 are capable of producing neuroprotective effects, mediated by antioxidant effects and the regulation of neurotransmission, neuroendocrine signaling, and neurotrophic factors [140], resulting in the alleviation of anxiety symptoms [141].

### 5.19. Antihypertensive Effects

Hypertension is a progressive condition that can lead to serious problems, such as acute coronary syndromes, chronic cardiac failure, and stroke [142]. Inflammation, the dysregulation of the renin-angiotensin pathway, autoimmune vascular malfunction, and oxidative stress are among the pathophysiological etiologies associated with the development of hypertension [143]. The antihypertensive effects of *G. biloba* have been observed in a variety of animal models. In a study of rats with impaired kidneys, EGb 761 exerted hypotensive and renoprotective effects by inhibiting renal NO overproduction and reducing IL-6 and TNF-*α* levels in kidney tissue [99]. NO lowers blood pressure by dilating blood vessels, and the hypotensive effect of GBE may be due to increased eNOS expression and NO production. GBE also increases endothelial intracellular Ca^2+^ levels and endothelium-dependent vasodilation, leading to hypotension [59].

### 5.20. Neuroprotective Effects

The rapid progression of neurodegenerative diseases occurring within the general population has highlighted the increased need for research to identify the pathogenesis of neurodegenerative disease and develop alternative therapies. Excitatory toxicity is a primary cause of intracerebral neuron death. Neurodegenerative disorders may be prevented by enhancing the cerebral blood supply. *G. biloba* prevents pathological hyperactivity, allowing the body to reclaim its natural physiological equilibrium. In addition, terpenoid and flavonoids in *G. biloba* seeds have been shown to improve blood circulation in the brain [40]. GBE has been shown to improve a variety of ailments including AD and brain ischemia, two age-related neurodegenerative diseases that are important causes of death and morbidity among older adults [144]. The increased occurrence of cerebrovascular disease has been associated with chronic inflammatory disorders [145]. AD is a neurological disease affecting people as they age and results in cognitive impairment [146]. Both immune components in diazinon-exposed fish in control and those fed 0.5 and 4 g GB/kg diets reduced significantly throughout the exposure. Immunity components in fish fed 1 and 2 g GB/kg diets did not change over the exposure period. Furthermore, diazinon increased the expression of the IL-1 and TGF-1 genes in fish fed 0.5 and 4 g GB/kg diets. Finally, at optimal dietary levels (1-2 g GB/kg diet), GB has an immunogenic effect against diazinon toxicity. However, GB demonstrated immunosuppressive effects at high dietary levels (4 g GB/kg intake), indicating that its levels in the diet should be optimized [100].

## 6. Commercial Use

*G. biloba* leaves are used in traditional Chinese medicine to treat neurological problems, circulatory disorders, and respiratory diseases. *G. biloba* leaves are also used as insecticides and fertilizers [147]. *G. biloba* leaf extracts are used as food additives due to potential medicinal benefits, which have captivated the global market due to the potential to improve wellbeing [148]. Pharmaceutically bioactive compounds have been identified in *G. biloba* leaf extracts, including glycosides and ginkgolides. EGb 761 is a common extract used in several countries, including the United States, Germany, China, and France, and is marketed in Europe as a drug for the treatment of cardiovascular disease. EGb 761 is distributed as a dietary supplement to the diet under the trade name Ginkgold. The use of *G. biloba* components, such as ginkgolides A, B, C, J, and M, is increasing at a rapid pace [4]. *Ginkgo* shells, which are produced as industrial by-products at a high annual rate, have not been thoroughly studied. Lignin is an abundant component of *Ginkgo* shells, including high yields of ferulates, p-coumarates, and vanillin, and is found at higher levels than in most softwoods and hardwoods, making *Ginkgo* shells a useful source for food and drink flavoring agents. It also has excellent antioxidant functions, with an optimum radical scavenging index of 6.9, higher than industrial antioxidants butyl hydroxyanisole and butylated hydroxytoluene [149]. *Ginkgo* nuts have historically been used to treat coughs, sputum, fever, diarrhea, toothaches, skin diseases, gonorrhea, and overactive bladder. *Ginkgo* nuts have also been eaten as a side dish [147]. *G. biloba* seeds have a hypocholesterolemic effect on the lipid metabolism [150], and EGb 761 has been shown to inhibit diabetic-induced cataracts under rat lenses cultured in high-glucose conditions [82].

## 7. Synergistic Effects

Synergism is the phenomenon in which treatment with two or more agents simultaneously result in more influential health outcomes than would be expected from the sum of the individual effects [151]. By regulating ROS production and lectin-type oxidized LDL receptor 1 (LOX-1) and p38 MAPK expression in human ex vivo endothelial artery cells, therapeutic doses of GBE and aspirin have been demonstrated to have synergistic effects reducing oxidative stress induced by activated platelets [152]. GBE inhibited platelet aggregation induced by tertbutyl hydroperoxide and Fe^2+^ in a dose-dependent manner. These findings suggest that GBE can inhibit oxidative stress-stimulated platelet aggregation in the brain and myocardial systems [153]. Polyprenols isolated from lipids are newly identified components found in *G. biloba* leaves. Petroleum ether extraction, saponification, and molecular distillation were used to extract *Ginkgo* lipids. Previous investigations have examined the antibacterial and antifungal properties of GBP and the chemicals isolated from GBL lipids against *Staphylococcus aureus*, *Aspergillus niger*, or *Salmonella enterica* and their synergistic interactions. The highest efficacy was identified for nerolidol, and the combination of GBP and isophytol exhibited the best synergistic activity against *Salmonella enterica* among the three strains examined [154]. RCTs have examined the use of GBE for periods ranging from 2 weeks to 2 years, with dosages varying from 80 to 720 mg/d. Interactions have been identified with inhibitors of monoamine oxidase, alprazolam, haloperidol, warfarin, and nifedipine [155]. The combination of isorhamnetin and caffeic acid facilitates the expression of CAT and SOD enzymes in *E. coli*, which may be due to the synergistic effects between C-3 and EGb 761 [156]. The formulation of green tea and GBE has shown success for the improvement of skin conditions, and the FBlend solution results in more pronounced improvements in skin elasticity [157].

## 8. Adverse Effects

Although *G. biloba* has the potential to treat a variety of diseases, some drawbacks have also been identified. Restani et al. recently explored the negative impacts of plant food additives and botanical preparations and observed that 39 plants species, representative of 59% of all plants tested, had negative impacts on people. The fourth most hazardous species was determined to be *G. biloba* [158].

### 8.1. *G. biloba* Seeds

Tonic and clonic convulsions, vomiting, and loss of consciousness are symptoms associated with *G. biloba* seed poisoning. The neurotoxic chemical MPN, also known as ginkgotoxin, and its derivative MPN glucoside are the primary sources of toxicity. MPN is structurally similar to vitamin B6 and hinders the synthesis, metabolism, and activities of vitamin B6 [159]. MPN inhibits the enzymatic activation of vitamin B6 by pyridoxal kinase, resulting in vitamin B6 deficiency and decreased *γ*-aminobutyric acid (GABA) synthesis. MPN values in *G. biloba* seeds range from 170 to 404 ppm. A 23-month-old child in Switzerland suffered two afebrile tonic-clonic seizures after swallowing an unknown quantity of *G. biloba* seeds, and the toxicity was verified by monitoring MPN levels in both blood and urine [160]. A 51-year-old Korean woman who ingested 1 kg of *G. biloba* nuts within 1 hour experienced tonic-clonic seizures 12 hours later, associated with postictal confusion and a serum vitamin B6 level of 2.2 g/L [161].

### 8.2. *G. biloba* Leaf Extracts

Using modern methodologies, MPN identified in *G. biloba* leaf extract is below the typical detection limit (9 ppm), although the potential that leaf extracts may be contaminated by *G. biloba* seeds or fruits cannot be entirely excluded. In individuals receiving medications metabolized by CYP2C19 and CYP2C9, GBE consumption should be monitored. More studies remain necessary to determine how MPN interacts with medications that are CYP3A4 or P-gp substrates [162]. Standardized *G. biloba* leaf extract has been examined for the potential to increase the risk of bleeding [163], and the literature regarding bleeding risks has been contentious, although additional case studies continue to be released [164, 165]. When combined with warfarin, *G. biloba* leaf extract significantly reduced the risk of bleeding, according to one study of a huge medical record repository in the US [166]. Other *G. biloba* leaf extract adverse effects have also been recorded. For example, a 35-year-old woman experienced irregular nocturnal palpitations that were connected to the proarrhythmic activities of *G. biloba*, and a 66-year-old woman experienced allergic contact dermatitis after contact with *Ginkgo* tree fruit amid *Ginkgo* leaves [167, 168].

## 9. Toxicity

High doses and long-term administration of *G. biloba* can cause several toxic effects, as demonstrated in both in vivo and in vitro studies.

### 9.1. In Vivo Toxicity

*G. biloba* toxicity has indeed been studied in vivo in rats, in addition to clinical trials. The evaluation of genotoxic and carcinogenic potential represents significant toxicity endpoints during the safety assessment of a drug [169]. In rats and mice of both sexes, a three-month *G. biloba* treatment increased liver weights and the rate of hepatocytic hypertrophy, and male rats presented with a considerably increased frequency of hepatocellular adenomas. If the dose is continued for 2 years, the survival rate for both male rats and female mice decreased. Another hepatogenesis mechanistic analysis revealed that substantial variations in molecular changes were found between spontaneous and ginkgo-induced HCC in B6C3F1 mice, although these carcinomas were morphologically indistinguishable [170]. The expression of genes associated with Nrf2-mediated oxidative stress and drug metabolic enzymes in the Myc gene-centered network termed “cell cycle, cancer, and cellular mobility” were found to differ significantly in the livers of male B6C3F1 mice based on microarray analysis [171]. During pregnancy, the treatment of female mice with *G. biloba* capsules at a dose of 100 mg/kg/day resulted in the significant incidence of abnormalities in mouse fetuses [172]. Polyprenols are common active lipids with neither acute nor subchronic toxicity in rats, which have been found in 0.35% of dried *Ginkgo* leaves. The toxicity of *G. biloba* was less evident during pregnancy. A standardized EGb 761 extract with specified active component concentrations of ginkgolic acids had no influence on embryo-fetal development, preimplantation, or postimplantation losses in CD-1 mice fed 100–1,225 mg/kg/day EGb 761 daily from day 6 to day 15 of pregnancy [162, 173].

### 9.2. In Vitro Toxicity

The National Toxicology Program (NTP) assessed the genotoxicity of *G. biloba* leaf extract containing comparable components of known items using bacterial gene mutation tests. Extract (1–10 mg/plate) was mutagenic in *Salmonella typhimurium* strains TA98 and TA100 and in the *E. coli* strain WP2 uvrA/pKM101 [174]. The extract also increased intracellular ROS while decreasing GSH levels [175]. At a concentration of 40 M concentration, kaempferol and quercetin inhibit cellular growth in oral cancer cell lines [176]. Picrotoxin, a well-known GABA_A_ receptor antagonist, resembles ginkgolides [177]. The resazurin reduction test revealed that three large ginkgolic acids with different alkyl or alkenyl groups (13 : 0, 15 : 1, and 17 : 1) were cytotoxic at 50 M in male Chinese hamster lung fibroblast-like V79 cells.

## 10. Clinical Studies

Clinical trials refer to experimental processes conducted on humans to determine the efficacy and safety of bioactive compounds, such as those found in *G. biloba*. Cai et al. observed that the maximal combination of 240 mg EGb (QD) and 400 mg sorafenib (BID) was effective and tolerable for HCC patients in a clinical trial assessing the safety and efficacy combined treatment for patients with advanced HCC [178]. Clinical studies examining 240 mg daily EGb 761 administration to dementia patients have indicated that EGb 761 can stabilize or delay mental functional decline, especially among individuals with neuropsychiatric symptoms [179]. A clinical experiment was conducted to examine the antidiabetic efficacy of GBE when combined with metformin as adjuvant therapy. In a 3-month controlled, multicenter, double-blinded study, 60 T2DM patients were randomly assigned to one of two classes (GBE 120 mg/day or placebo (starch) 120 mg/day). The administration of GBE dramatically decreased fasting plasma glucose levels, insulin levels, blood HbA1c values, waist circumference, body mass index, and visceral adiposity index [180]. Increased very-low-density lipoprotein (VLDL) secretion by liver cells leads to insulin tolerance and hyperlipidemia [59]. Adjuvant *G. biloba* treatment improved total cholesterol (median: 0.61 mmol/L; 95% confidence interval (CI) 0.33–0.90 mmol/L), LDL-C (median: 0.32 mmol/L; 95% CI 0.16–0.48 mmol/L), HDL-C (median: 0.26 mmol/L; 95% CI 0.15–0.37 mmol/L), and TG (median: 0.32 mmol/L; 95% CI 0.20–0.43 mmol/L) levels compared with statin therapy [181]. The therapeutic efficacy of EGb 716 as an antioxidant with free radical scavenging properties was explored in an RCT, in which the Abnormal Involuntary Movement Scale was used to quantify the severity of TD symptoms as the primary outcome. EGb 716 (240 mg/day) outperformed the placebo group for minimizing the incidence of TD, and a meta-analysis suggested that adjunctive EGb 716 improved the incidence of TD among schizophrenia patients [133]. Patients with GAD (25–30 patients per group) treated with EGb 761(240 mg or 480 mg placebo for 4 weeks) displayed increased cognitive abilities and reduced anxiety [136]. Flavonoids, lactones, kaempferol 3-O-?-D-glucopyranoside, isorhamnetin-3-O-glucoside, myricetin, ginkgolide A, and bilobalide in *G. biloba* have displayed antiaging effects in human dermal fibroblasts (HDFs) through reduced ROS production and matrix metalloproteinase 1 (MMP-1) degradation. EGb tablets (19.2 mg per dose, 3 times a day) showed antidepressant effects in 136 older adult patients with depression through the modulation of serotonergic and dopaminergic neurotransmission [116] (Table 4).

## 11. Conclusion and Future Perspectives

*G. biloba* has been studied in a number of RCTs and has consistently demonstrated positive safety profiles and promising results for the treatment of a wide range of symptoms and diseases. Various tests have been conducted in both animal models and humans, and the underlying mechanisms of action have been defined. However, potential drug reactions and adverse effects are still being investigated in clinical trials. The uses of diverse methodologies, metrics, and research methods across studies have complicated the determination of effectiveness and the ability to perform cross-study comparisons. As a result, more sensitive and systematic outcome measures should be developed, in addition to the validation of interrater and intrarater reliabilities, concurrent test formats, test-retest reliability, and more accurate patient descriptions and diagnoses, to improve the ability to compare the outcomes of randomized studies. Future studies should discuss the fact that the doses used in many animal studies are not indicative of human dose levels. Reactions to *G. biloba* are influenced by various factors, including pharmacology, pharmacokinetics, pharmacodynamics, bioavailability, washout times, and long-term consequences of individual components. Dose-response properties, optimal pacing and length of clinical treatments, functions as an adjunctive therapy treatment, conditions for which *G. biloba* treatment is the most and least useful, side effects, and drug reactions have been excluded from the scope of this review; however, these factors are significant and deserve further research. Moreover, a wide variety of potential therapeutic effects for *G. biloba* such as protection of DNA from oxidative harm and mitochondrial dysfunction, the inhibition of mitochondrial ROS and apoptosis (mediated by mitochondria, caspases, and death receptors), function in the mitochondrial respiratory chain, mitochondrial DNA (mtDNA) effects, regulation of mitochondrial membrane potential, and intramitochondrial Ca^2+^ homeostasis have not yet been extensively studied.

In conclusion, *G. biloba* can serve as a natural substitute and complementary method for the treatment of a wide range of symptoms and disorders. When physicians and researchers are knowledgeable about the structure, indications, conditions, dosages/durations, pathways, concentration-dependent consequences and effectiveness, adverse reactions, and potential drug interactions, they can make decisions for improved patient experiences, safety, and analysis.

## Figures and Tables

**Table 1:** Nutraceutical value of *Ginkgo biloba* [45].

Main nutrition facts	Amount per 100 g
Sodium	7 mg
Potassium	510 mg
Calcium	2 mg
Saturated fatty acids	0.319 g
Calories	182 Kcal (761.49 kj)
Calories from fat	14.0616 Kcal (58.83 kj)
Cholesterol	0 mg
Food energy	Amount per 100 g
Calories	182 Kcal (761.49 kj)
Calories from protein	14.9904 Kcal (62.82 kj)
Calories from carbohydrate	153.032 Kcal (640.29 kj)
Calories from fat	14.0616 Kcal (58.83 kj)
Fats and fatty acids	Amount per 100 g
Total fat	4.75 ± 0.22 g
Polyunsaturated fat	0.618 g
Monounsaturated fat	0.619 g
Saturated fat	0.319 g
Carbohydrates	Amount per 100 g
Carbohydrate by difference	72.98 ± 0.20 g
Protein and amino acids	Amount per 100 g
Protein	12.27 ± 0.24 g
Serine	0.29 g
Proline	0.34 g
Glycine	0.23 g
Leucine	0.31 g
Glutamic acid	0.83 g
Histidine	0.1 g
Valine	0.28 g
Phenylalanine	0.17 g
Arginine	0.42 g
Cystine	0.02 g
Alanine	0.24 g
Methionine	0.05 g
Aspartic acid	0.54 g
Lysine	0.2 g
Isoleucine	0.2 g
Threonine	0.26 g
Tryptophan	0.07 g
Tyrosine	0.06 g
Vitamins	Amount per 100 g
Vitamin C	15 mg
Vitamin B1	0.22 mg
Vitamin B2	0.09 mg
Vitamin B3	0.16 mg
Vitamin B6	0.328 mg
Total folate	54 *µ*g
Vitamin B9	0 *µ*g
Folate (dietary folate equivalents)	54 *µ*g
Food folate	54 *µ*g
Vitamin B12	0 *µ*g
Vitamin A	558 iu
Vitamin a (retinol activity equivalents)	28 *µ*g
Retinol	0 *µ*g
Vitamin D	0 iu
Vitamin D	0 *µ*g
Minerals	Amount per 100 g
Manganese	0.113 mg
Calcium	2 mg
Copper	0.274 mg
Iron	1 mg
Zinc	0.34 mg
Magnesium	27 mg
Sodium	7 mg
Phosphorus	124 mg
Potassium	510 mg
Sterols	Amount per 100 g
Cholesterol	0 mg
Other nutrients	Amount per 100 g
Ash	10.01 ± 0.06 g
Water	55.2 g

**Table 2:** Major bioactive components of *Ginkgo biloba*.

Class	Plant parts	Major chemical constituents	Bioactivity	References
Polyprenols	Leaf	Di-trans-poly-cis-octadecaprenol	Antibacterial properties and safety against the attack by A*β*25-35	[46]
Flavonoids	Leaf	Quercetin (c), kaempferol (d), isorhamnetin (e), rutin, luteolin, delphidenon, myricetin	Antioxidation, anticancer, antibacterial, antiviral, anti-inflammatory, and neuroprotective effects	[47]
Organic acids	Leaf	Benzoic acid derivatives (ginkgolic acid), N-containing acids	Inhibitory effects on xanthine oxidase (XOD) and antitumor properties	[48]
Biflavonoids	Leaf	Sciadopitysin, ginkgetin, isoginkgetin, amentoflavone, bilobetin, 5′-methoxybilobetin	Antiadipogenesis and antiobesity properties and significant inhibitory effects on thrombin activity	[49]
Terpenoids	Root	Triterpenes: sterols	Protective effects to cerebral hippocampal neurons from epilepsy, antioxidant, anti-inflammatory, antiplatelet, antilipidemic and antiapoptotic properties, enhanced memory and learning abilities, and reduced neuronal damage	[50, 51]
	Leaf/root/bark	Sesquiterpene: bilobalide (b)		
	Leaf/root/bark	Diterpenes: ginkgolides *M* (a)		
	Leaf/root/bark	Diterpenes: ginkgolides A, B, C, and J (a)		
Others	Leaf	Waxes, steroids, 2-hexenal, cardanols, sugars, catechins, proanthocyanidins, phenols, aliphatic acids, rhamnose	Antioxidant, anti-inflammatory, antidiabetic, antiapoptotic, antiradiation, antiviral, antitumor, hepatoprotective, and antiatherosclerosis pharmacological properties	[52–58]

**Table 3:** In vivo and in vitro studies on therapeutic effects of *Ginkgo biloba*.

Field of study	Bioactive compounds	Subject	Dosage	Outcome	Mechanism of action	References
Treatment of respiratory diseases	Ginkgetin, biflavones	In vitro, A549 cells	0.01, 0.1, 1, 10, and 40 mg/kg	↓IL-4, ↓IL-5, ↓IL-6, ↓IL-8,↓IL-13, ↓TNF	Ginkgetin significantly reduced irregular expression of the Akt and p38 pathway, whereas biflavones reduced MUC5AC mRNA expression	[71]
		In vivo, an allergic mouse model				
	Ginkgolide B (GB)	In vitro, RAW 264.7 cell culture model	10, 20 or 40 mg/kg	↓LPS-induced IL-1 mRNA and protein levels, ↓IL-10 protein level, ↓exudation of plasma protein, ↓myeloperoxidase activities	GB and GM were shown to be a key component in the treatment of lipopolysaccharide-induced lung injury	[70]
	Ginkgolides mixture (GM)	In vivo, mouse model	19.1, 38.2 or 76.4 mg/kg			
Anticancer effects	GBE	In vivo, rats	1 kg/2000 mL	↑ING-3 gene expression, ↓FOXP1 gene expression, ↓AFP, ↓GPC-3, and ↓CEA	Treatment with *Ginkgo biloba* leaf extracts resulted in a substantial decrease in serum tumor markers and oncogene downregulation, as well as upregulation of a tumor suppressor gene in liver tissue	[72]
	GBEE	In vitro, C57BL/6J mice	5, 10, 20, 40, 80 and 160 *µ*g/mL	↓*β*-Catenin expression, ↓p-Akt/Akt expression, VEGF and VEGFR expression, ↓mRNA levels of VEGF and VEGFR2	GBEE demonstrated the antitumor and antimetastatic actions due to the induction of the Wnt/*β*-catenin-VEGF signaling pathway, which inhibited tumor angiogenesis	[73]
	GBE	In vitro, gastric carcinoma SGC7901 cells	100, 200, 300, 400 mg/L	↑G1 stage cell percentage, ↓S stage cell percentage, ↓mRNA and protein for the cyclin D1 and c-myc genes	GBE inhibited the proliferation of gastric carcinoma SGC7901 cells	[74]
	GBE	In vitro, gastric cancer cells	GBE with 0.9% NaCl	Suppressed and inhibited expression of KSR1, p-KSR1, ERK1/2, and p-ERK1/2, ↓MDA, ↑GSH-Px, ↑SOD	*Ginkgo biloba* extract improved chemotherapy sensitivity and reversed chemoresistance in gastric cancer cells by suppressing the KSR1-mediated ERK1/2 pathway	[92]
Antidiabetic effect	Ginkgolide B	In vivo, rats	5 mg^−1^ kg^−1^ day^−1^	↑SOD and eNOS operations, ↑H_2_S processing, ↓MDA, ↑mRNA expression of GPX1, ↑protein expressions of CBS and CGL, ↑mRNA, expression of GPX1	Ginkgolide B reduced the progression of endothelial and vascular dysfunction in diabetic rats by increasing antioxidants and enhancing vascular control, and by increasing the expression of CBS and CSE, it prevented the development of vascular dysfunction	[35]
	Bilobalide	In vitro, 3 T3-L1 preadipocytes	10, 20, and 50 *μ*M	↓Adipokine secretion, ↓NF-ĸB/JNK activation, ↑adiponectin	Bilobalide lowered the risk of developing type 2 diabetes by protecting against hypoxia-induced inflammation and other complications	[77]
Antiobesity effects	GBE	In vivo, rats	500 mg/kg	↓Nutrition/energy consumption, ↑Adipo R1 and ↓IL-10 gene expressions, ↑IR and Akt phosphorylation, ↓BW, ↓TNF-stages, ↓NF-ĸB p65 phosphorylation	Repeated therapy with GBE resulted in substantial visceral adiposity deficiency, improved insulin sensitivity, reduced dyslipidemia, and activated the insulin signaling cascade.	[78]
	Ginkgolide B	In vivo, C57BL/6 mice	0.1 g/100 g	↑mRNA expression of PXR	Ginkgolide B therapy reduced body weight gain and increased hypertriglyceridemia	[80]
	Ginkgolide C	In vitro, HepG2 hepatocytes	3–100 *μ*M	↑Lipolysis, ↓lipid aggregation, ↑mRNA expression of ATL	Ginkgolide C decreased oleic acid-induced lipid aggregation	[80]
	Isoginkgetin, bilobetin, ginkgetin, and sciadopitysin	In vitro, pancreatic lipase	2.90 *μ*M to 12.78 *μ*M	Inhibited pancreatic lipase	Biflavones in *Ginkgo biloba* including isoginkgetin, bilobetin, ginkgetin, and sciadopitysin had high to moderate inhibitory effects on PL	[44]
Antilipidemic effects	GBE	In vivo, rabbits (p.o for 6 weeks)	10 mg^˗1^ kg^−1^ day	↓TC, ↓TG, ↑HDL-C, ↓MDA, ↑GSH	*Ginkgo biloba* slowed the development of atherosclerosis by altering the lipid profile and suppressing the inflammatory response and oxidative stress.	[81]
Treatment of cardiovascular diseases	*Ginkgo* seed ethanol extract	In vivo high-fat diet (HFD) mice (p.o. for 4 weeks)	300 mg/kg/day	↓High-fat diet mice's epididymal adipose tissue weight and adipocyte size, ↓high-density lipoprotein	Seeds of *Ginkgo biloba* provided a hypocholesterolemic effect by inhibiting lipid synthesis, which may be effective for the treatment of cardiovascular diseases	[82]
	Detoxified *Ginkgo* nut powder (DGP)	In vivo obese C57BL/6J mice	0.67 mg/kg ginkgolic acid, and 701.3 mg/kg gingko flavonoids	↓Liver and body fat weights, ↓serum and hepatic amounts of triglyceride, ↓low-density lipoprotein cholesterol, ↓total cholesterol, ↓leptin, ↑PPAR*α*, ↑hormone-sensitive lipase, ↑adipose triglyceride lipase, ↑monoacylglycerol lipase	Detoxified *Ginkgo biloba* nut powder minimized damage and increased the regular lipid metabolism	[83]
Antimicrobial effects	Ginkgolic acids	Two bacteria and four fungi	0.11 g/kg	*K. pneumoniae* had the maximum antimicrobial activity, with a MIC of 1.0 × 10^5^ g/mL, while *E. coli* had the lowest antimicrobial activity, with a MIC of 7.5 × 10^3^ g/mL.	Ginkgolic acids had a wide antimicrobial repertoire against bacteria such as *E. coli* and *Bacillus subtilis*, as well as fungi like *Penicillium purpurogenum*, *Penicillium camemberti*, and *Aspergillus niger*	[93]
Antioxidant effects	Ginkgolide B	In vitro, human neuroblastoma SH-SY5Y cells	0–100 *µ*M	↓ROS/RNS, ↑mitochondrial APE1 level, ↑OXPHOS complexes I and IV activity	Ginkgolide B restored the neuroprotective function of apurinic/apyrimidinic endonuclease 1 and mitochondrial oxidative phosphorylation against A25-35-induced neurotoxicity	[86]
	GBE	In vivo C57BL/6J male mice	20, 40, 80, or 100 mg/kg	↑Glutathione, ↑superoxide dismutase, ↓MDA, ↓NO, ↑phosphatase PP2A	GBE or similar drugs may be used as a long-term therapeutic alternative in elderly patients with ischemic brain injuries	[87]
	GBE	In vivo, cisplatin-administrated rats	100 mg/kg	↓NO, ↓GSH, did not affect the decreased MDA levels	GBE reduced cisplatin's oxidative stress-related neurological side effects	[94]
	GBE	In vivo, rats	Dissolved in 0.1% methyl alcohol	↓Intracellular ROS, ↓GSK3*β* phosphorylation, ↓GSK3*β*, ↓ROS	GBE demonstrated antioxidative activities by affecting the modulation of GSK3 to suppress zinc-induced tau phosphorylation at Ser262	[95]
	GBE	In vivo, aged female rats	100 mg mL^−1^	↓8-OHdG, ↓MDA, ↑BDNF levels, ↓locomotor activity, ↓anxiety levels	GBE supplementation increased cognitive functions by reducing oxidative damage and increasing BDNF levels	[96]
*Ginkgo biloba* in wound healing	GBE	In vivo, Sprague Dawley rats	40 mg/kg/day	↑TSSA, ↑NSSA, ↑GRD, ↑GST, ↓XO	GBE has strong antioxidant properties and is likely to be a valuable medication for gamma-irradiation safety and/or as an antioxidant against oxidative stress	[89]
	GBE	In vitro, normal human keratinocytes (NHKs)	5 kg of fresh leaves dissolved in 0.1–1% concentrated fresh medium	↓VEGF, ↓CXCL8/IL-8 levels	GBE, alone or in combination with EGCG, led to mild inflammation processes in angiogenesis-related skin diseases	[90]
	GBE	In vivo, Fisher rats	200, 100, and 50 mg/kg	↓Wound surface area, ↓4-hydroxy-2-nonenal-stained cells, ↓8-hydroxy-2′ deoxyguanosine-stained cells	Extract of *Ginkgo biloba* had a preventive function against frostbite and likely alleviated reperfusion damage by minimizing tissue peroxidation	[91]
Antiplatelet activity	Ginkgolide A	In vitro, human umbilical vein endothelial cells (HUVECs)	10, 15 and 20 *μ*M	IL-4, IL-6, IL-13, signal transducer, and activator of transcription-3 (STAT3) phosphorylation were substantially inhibited	Ginkgolide a demonstrated STAT3-mediated inhibition of inflammatory response in high-glucose-stimulated human umbilical vein endothelial cells	[5]
	Ginkgolide B	In vitro, freshly human platelets	0.6 mg/ml	↓PF4 and CD40L expression, ↓phosphorylation of Syk and p38MAP, ↓Ca^+2^efflux	Ginkgolide B prevented platelet release by inhibiting the phosphorylation of Syk and p38 MAPK in thrombin-stimulated platelets	[97]
	*Ginkgo* terpenoids	In vitro, Sprague-Dawley rats	27.69% bilobalide, 37.15% ginkgolide A, 22.04% ginkgolide B, and 11.66% ginkgolide C	The AUC and C_Max_ of GC are smaller than those of the other ginkgolides, and it is methylated in vivo much faster than the other GBE constituents	Oral *Ginkgo* terpene lactone (GTL) doses for fasted and female subjects should be smaller than for fed and male subjects, respectively	[97]
	Bilobalide	In vitro, 3T3-L1 adipocytes	10, 20 and 50 *µμ* M	↑HIF-1 *αα* expression, ↑lactate and glycerol release, ↑ROS production, ↑lipid and protein oxidation, ↓superoxide dismutase, ↓catalase, ↑TNF-*αα*, ↑IL-6, ↑IL-1 *ßβ*, ↑IFN-*γ*	Bilobalide lowered hypoxia-induced oxidative stress, inflammation, and mitochondrial dysfunction in 3T3-L1 adipocytes	[98]
Antihypertensive effects	GBE	In vivo, rats	100 mg−1 kg−1 day−1	↑GSH, ↓nitrite levels, ↑protein expressions of iNOS, TNF-*α*, IL-6, and IL-1*β* in the kidney, nitrite levels, ↓MDA, ↑mRNA eNOS expression, ↓BP	The standardized extract of *Ginkgo biloba* provided the protective effect against hypertension with hypercholesterolemia-induced renal damage	[99]
Neuroprotective effects	GBE	In vivo, APP/PS1 transgenic mouse model of AD	50 mg/kg	↓Insoluble amyloid beta (a*β*), ↓proinflammatory cytokines, ↓inducible nitric oxide synthase (iNOS), ↑ anti-inflammatory cytokines, ↑arginase-1 (Arg-1)	GBE protected the APP/PS1 mouse from damage by regulating inflammation in the brain	[100]
	GBE and donepezil	In vivo, aged rats	GBE (100 mg/kg/day), donepezil (1.5 mg/kg/day)	↑Basal ACh levels, ↓AChE, ↓HACU ↓ extracellular choline release, ↑ChAT activity	GBE and donepezil combination demonstrated neuroprotective properties by lowering choline levels	[101]
Antiaging effect	Flavonoids, lactones, kaempferol 3-O-*β*-D-glucopyranoside, isorhamnetin-3-O-glucoside, myricetin, ginkgolide A, and bilobalide	In vitro, human dermal fibroblasts (HDFs)	n.m.	Improved skin condition and antiaging effect	*Ginkgo biloba* extraction impaired ROS and MMP-1 degradation in HDFs because of elevated levels of flavonoids and lactones	[25]
Immunomodulatory effects	*Ginkgo biloba* in powder form	In vivo, rainbow trout fish	In non-diazinon fish, 1 and 2 g GB/kg diet for 60 days. In diazinon-exposed fish, 0.5 and 4 g GB/kg diet was fed	Enhanced the immunity at optimum dietary levels (1-2 g/kg diet) but showed immunosuppressive effects at high dietary levels (4 g/kg diet)	*Ginkgo biloba* increased monoamine neurotransmitter levels and altered the glyphosate-induced hepatotoxicity, nephrotoxicity, lipid peroxidation, and genotoxicity	[102]
Anti-inflammatory effect	*Ginkgo biloba*, ethyl acetate extract of *Ginkgo biloba*, the extract of total flavonol glycosides and full extract of *Ginkgo biloba* seed	In vivo, 28-day-old BALB/*c* mice (half females and half males)	10 mg/kg of body weight for low dosage and 40 mg/kg of body weight for high dosage form	Showed anti-inflammatory effect in airway inflammation	Ginkgetin reduced the expression of mRNA MUC5AC and regulates pathways p38 and Akt of the A549 cell model stimulating HNE	[71]
	Ethanol extract of *Ginkgo* flowers, bilobetin, isoginkgetin	In vitro, RAW264.7 murine macrophage cell	Different extractive fractions at 10 *μ*g/ml or 100 *μ*g/ml, 24 h incubation, at a concentration of 100 *μ*M	Effectively inhibited the generation of proinflammatory cytokines and mediators, making them valuable for the treatment of inflammatory disease	Bilobetin and isoginkgetin can dramatically downregulate the levels of NO, TNF-*α*, IL-6, PGE2, iNOS mRNA, and COX‐2 mRNA, in a dose‐dependent manner, which may be the mechanism of their anti‐inflammatory effects	[103]
Hepatoprotective effect	*Ginkgo biloba* extract	In vivo, male SD rats	For 7 days, 60, 120, and 180 mg/kg daily dose before MTX treatment); silymarin (followed by MTX treatment)	Showed antioxidant and anti-inflammatory effects in methotrexate (MTX) induce marked hepatic injury	*Ginkgo biloba* extract reduced expression levels of TNF-*α*, p-JNK, caspase-3, and COX-2 pathways in rat liver	[104]
Anti-depressant effect	Diterpene ginkgolides (DGs)	In vivo, mice	DG (12.18 mg/kg) and venlafaxine (16 mg/kg) for 14 days	Exhibited antidepressant-like, but not anxiolytic-like, effects	DG reduced depressive symptoms and produced neuroprotective effects on the prefrontal cortex by changing differential metabolites primarily involved in amino acid, energy, and lipid metabolism	[105]

**Table 4:** Clinical studies on therapeutic effects of *Ginkgo biloba*.

Field of study	Bioactive compound	Subject	Dosage	Outcome	Mechanism of action	Reference
Anticancer effects	GBE (extract of *Ginkgo biloba*)	Human patients	240 mg EGb (QD) and 400 mg sorafenib (BID)	The combined toxicity profile of GBE and sorafenib proved to be close to that of GBE and sorafenib monotherapy.	GBE in combination with sorafenib increased safety and tolerance in patients with advanced HCC	[178]
Antidementia effects	GBE	Human patients	240 mg/day	↓Loss in memory, function, behavior, and global shift	GBE increased cognitive function, ADLs, and CGIC and decreased neuropsychiatric symptoms with statistical superiority in patients with cognitive dysfunction and dementia	[179]
	EGb761	Human patients	n.m.	↑Cognition and ↓neuropsychiatric symptoms	*Ginkgo biloba* was found to be more beneficial than a placebo in the treatment of dementia	[182]
	EGb 761	Human patients	120 mg/day	↑Cognitive function	In comparison to the placebo group, those who were given EGb 761 saw a substantial increase in cognitive performance.	[183]
	EGb 761	Human patients	240 mg/day	↑Cognitive function ADLs	In contrast to the placebo group, EGb 761 improved cognitive performance markedly.	[184]
	EGb 761	Human patients	120 mg/day; 240 mg/day	↑Cognition and ↓neuropsychiatric symptoms	In a subset of individuals with neuropsychiatric symptoms, the EGb 761 group outperformed the placebo group.	[185]
	EGb 761	Human patients	240 mg/day	↑Cognition and ↓neuropsychiatric symptoms	In opposed to the placebo group, the EGb 761 group showed considerable improvement in all clinical parameters, including neuropsychiatric symptoms.	[186, 187]
	GBE (extract of gingko biloba)	189 patients	EGb (240 mg/day) or donepezil (5 or 10 mg/day)	EGb 761, which is used to treat dementia in people with Alzheimer's disease, has similar effects on cognitive symptoms as donepezil, and it is less dangerous than donepezil.	n.m.	[188]
	GBE (extract of gingko biloba)	400 patients	240 mg/day	EGb 761 was found to be better than a placebo for people with dementia who had neuropsychiatric symptoms	EGb 761 is a polyvalent radical scavenger that helps mitochondria work better. It decreases blood viscosity, improves microperfusion, stops the formation of synaptotoxic A-oligomer, and reduces the toxicity of amyloid.	[189]
Antidiabetic effects	GBE	Human, T2DM patients	120 mg/day	↓HbA1c, ↓FBG, ↓plasma insulin, ↓BMI, ↓waist circumference, ↓VAI	GBE primarily improved the glycemic control through the suppression of release of glucose from the liver	[180]
Treatment of tardive dyskinesia	GBE (extract of gingko biloba)	3 human randomized controlled trial (RCT)	240 mg/day, 12 weeks duration	Reduced the severity of TD and clinical symptoms.	GBE scavenges free radicals and inhibited the formation of free radicals that reduce oxidative stress, enhance BDNF levels, and decrease the chance of neurotoxicity	[133]
Treatment of generalized anxiety disorder (GAD)	GBE	Patients with GAD (25–30 patients per group)	240 mg and 480 mg placebo for four weeks	Improve cognitive functions and reduce anxiety under pathological conditions	Flavonoids in GBE demonstrated powerful antioxidant qualities which helped to reduce anxiety-like disorders	[136]
Antilipidemic effects	*Ginkgo biloba* leaves	Human patients	A variety of doses	↓TC, ↑HDL-C, ↓LDL-C, ↓TG	*Ginkgo biloba* leaves were more effective than statin treatment alone in improving blood lipid parameters	[181]
Antidepressant effect	*Ginkgo biloba* extract (GBE)	136 depressed elderly patients	19.2 mg per time and 3 times a day	Improved depressive symptoms and decreased serum S100B expression.	GBE restored neurologic function and shows the antidepressant-like effect via modulation of serotonergic and dopaminergic neurotransmission	[116]
Treatment of vitiligo vulgaris	*Ginkgo biloba* BID	12 participants between 12 and 35 years old	60 mg of standardized *G. biloba* two times per day for 12 weeks	*G. biloba* has significant improvement in total vitiligo area scoring index and vitiligo measures and vitiligo European Task force spread	Having anti-inflammatory, immunomodulatory, and antioxidant properties, *Ginkgo* and its constituents impact the oxidative stress mechanisms of vitiligo decreasing basal corticosterone secretion, corticotrophin releasing hormone (CRH), and arginine vasopressin (AVP) gene expression	[189]
Treatment of ADHD	*Ginkgo biloba* extract (GBE)	50 patients	Ginko T.D.™ at a dose of 80–120 mg/day for 6 weeks	*G. biloba* would be beneficial for treatment of ADHD	n.m.	[190]

## Data Availability

The data used to support the findings of this study are included within the article.

## Abbreviations

*G. biloba*: *Ginkgo biloba*
AchE: Acetylcholinesterase
Arg-1: Arginase-1
Ach: Acetylcholine
ADL: Adenylosuccinate lyase
AFP: Alpha-fetoprotein
APE1: Apurinic/apyrimidinic endonuclease 1
ATL: Atlastin 1
BMI: Body mass index
BP: Blood pressure
BDNF: Brain-derived neurotrophic factor
ChAT: Choline acetyltransferase
COX: Cyclooxygenase
CXCL8: Chemokine (C-X-C motif) ligand 8
CD40L: Cluster of differentiation 40 ligand
CEA: Carcinoembryonic antigen
CBS: Cystathione beta synthase
CSE: Chromosome segregation
c-myc: Cellular myelocytomatosis
EGCG: Epigallocatechin-3-gallate
eNOS: Endothelial nitric oxide synthase
ERK1/2: Extracellular signal-regulated kinase1/2
FBG: Fasting blood glucose
FOXP1: Forkhead box protein P1
GSK3*β*: Glycogen synthase kinase-3-beta
8-OHdG: 8-Hydroxy-2'-deoxyguanosine
GRD: Glutathione reductase
GST: Glutathione-S-transferase
GB: Ginkgolide B
GM: Ginkgolides mixture
GPC-3: Glypican-3
GBEE: *Ginkgo biloba* exocarp extracts
GBE: *Ginkgo biloba* extract
GTL: Ginkgo terpene lactone
GC: Ginkgolide C
GSH: Glutathione
GPX1: Glutathione peroxidase 1
HCC: Hepatocellular carcinoma
HNE: Human neutrophil elastase
HbA1c: Glycohemoglobin
HACU: High affinity choline uptake
HIF-1*α*: Hypoxia inducible factor 1-alpha
HDL-C: High-density lipoprotein cholesterol
IFN-*γ*: Interferon gamma
ING-3: Inhibitor of growth 3
IL: Interleukin
LDL-C: Low-density lipoprotein cholesterol
JNK: c-Jun N-terminal kinase
LPS: Lipopolysaccharide
MMP-1: Matrix metallo proteinase-1
MUC5AC: Mucin 5AC
MIC: Minimum inhibitory concentration
MDA: Malondialdehyde
NSSA: Nonenzymatic superoxide scavenger activity
n.m.: Not mentioned
NF-ĸB: Nuclear factor kappa light chain enhancer of activated B cells
OXPHOS: Oxidative phosphorylation
PP2A: Protein phosphatase 2
PPAR*α*: Peroxisome proliferator-activated receptor
PXR: Pregnane *X* receptor
PL: Pancreatic lipase
P38MAPK: p38 mitogen-activated protein kinase
p-KSR1/KSR1: Kinase suppressor of Ras 1
PF4: Platelet factor 4
p-Akt/Akt: Protein kinase B (PKB)
ROS: Reactive oxygen species
RNS: Reactive nitrogen species
STAT3: Signal transducer activator of transcription-3
Syk: Spleen tyrosine kinase
SOD: Superoxide dismutase
TG: Triglycerides
TC: Total count of WBC
TSSA: Total (enzymatic and nonenzymatic) superoxide scavenger activity
TNF: Tumor necrosis factor
VAI: Visceral adiposity index
VEGF: Vascular endothelial growth factor
VEGFR2: Vascular endothelial growth factor receptor 2
Wnt/*β*-catenin: Wingless-related integration site beta-catechin
XO: Xanthine oxidase.
